# Plasmon-Enhanced Sunlight Harvesting in Thin-Film Solar Cell by Randomly Distributed Nanoparticle Array

**DOI:** 10.3390/ma14061380

**Published:** 2021-03-12

**Authors:** Marwa M. Tharwat, Ashwag Almalki, Amr M. Mahros

**Affiliations:** 1 Department of Electrical & Computer Engineering, King Abdulaziz University, Jeddah 21589, Saudi Arabia; mmzahran1@kau.edu.sa; 2 Physics Department, University of Jeddah, Jeddah 23218, Saudi Arabia; aelaryan@uj.edu.sa; 3 Department of Engineering Physics, Alexandria University, Alexandria 11432, Egypt

**Keywords:** FDTD, plasmonics, optical absorption

## Abstract

In this paper, a randomly distributed plasmonic aluminum nanoparticle array is introduced on the top surface of conventional GaAs thin-film solar cells to improve sunlight harvesting. The performance of such photovoltaic structures is determined through monitoring the modification of its absorbance due to changing its structural parameters. A single Al nanoparticle array is integrated over the antireflective layer to boost the absorption spectra in both visible and near-infra-red regimes. Furthermore, the planar density of the plasmonic layer is presented as a crucial parameter in studying and investigating the performance of the solar cells. Then, we have introduced a double Al nanoparticle array as an imperfection from the regular uniform single array as it has different size particles and various spatial distributions. The comparison of performances was established using the enhancement percentage in the absorption. The findings illustrate that the structural parameters of the reported solar cell, especially the planar density of the plasmonic layer, have significant impacts on tuning solar energy harvesting. Additionally, increasing the plasmonic planar density enhances the absorption in the visible region. On the other hand, the absorption in the near-infrared regime becomes worse, and vice versa.

## 1. Introduction

In the recent decade, the achievement of cheap, inexhaustible energy, and clean sources has been an area of great interest for many researchers all over the world [1]. The photovoltaic solar cell plays an important role among a wide range of renewable resources as an environmentally friendly energy source compared with fossil fuels [2]. However, the cost of solar cells needs to be lessened due to silicon as a bulk active material, processing, and packaging. The only way to eliminate this major difference in cost is by exchanging the silicon waver by thin films of semiconductors deposited on an inexpensive substrate such as plastic, sapphire, or stainless steel [3]. At the same time, we need to boost the efficiency of solar cells. 

In the present story, a huge part of the sunlight is misdirected and is not fully absorbed by the active layer and employed. This leads to a high cost of photovoltaic modules. Generally, absorption of light within the photoactive layer can be boosted by increasing the optical path length within the cell and reflection reduction on the front surface. A considerable amount of theoretical and experimental work has proposed a variety of light-trapping techniques, such as refractive index matching [4], surface texturing [5], diffraction gratings [6], antireflection coatings [7], photonic crystals [8], nanostructures [9], and metallic nanoparticles [10]. 

Plasmonic metamaterials have been recently recognized as a new border of engineering since the discovery of their confinement of optical field [11,12,13]. It has been found that the plasmonic effect induced by metamaterial absorbers is a promising approach to achieve light trapping in thin-film solar cells [10]. However, the fulfillment of broadband response metamaterial absorbers is quite challenging due to the inherent bandwidth limitations. Extensive studies have been reported to broaden the absorption spectrum of metamaterial absorbers, including elliptical nanowires [14], multiple resonators using in-plane arrangement [15], and multilayer stacking of resonant elements in the vertical direction [16] have been used. 

The incorporation of metallic nanoparticles can enhance light absorption and increase the optical path length by taking the advantage of localized surface plasmon excitation and light scattering for improving photon management and sunlight harvesting [17]. Besides, the active layer thickness is almost not affected by metallic nanoparticles which reduce overall cost. Metallic nanoparticles are introduced with various shapes [18], in different ways [19], and at several relative positions [10]. The efficient conversion of photocurrent has been reported by adding induced gold particles incorporated in a TiO_2_ matrix [20]. Later on, absorption enhancement is investigated using silver nanoparticles with different shapes such as hemispheres and cones [11]. Integration of the metallic gratings and an antireflection (AR) coating has been proposed for efficient absorption [6]. Using silver cubic nanoparticles to enhance the efficiency of PbS quantum dot/ZnO nanowire solar cells has also been proposed [21]. Unity absorption, in the visible and near-infrared regimes, has been investigated theoretically using gold ring nanoparticles and a back reflector. More recently, a silver double nanoparticles system separated by a small gap suspended onto a silicon substrate has been proposed to increase the coupling of photons into plasmonic modes [22]. 

On the other hand, the plasmonic effects could either ameliorate or afflict the performance. The scattering or absorbing of light by metal nanoparticles is very sensitive to the metal nanoparticles’ shape and size, the surrounding medium, and the surface coverage on the substrate [23]. As a result, still, it is a great challenge to improve the integrated solar cell efficiency. For that reason, one should design metallic nanoparticles to diminish absorption and boost the scattering across the desired frequency regime. It is worth mentioning that from the technical point of view, obtaining reproducible nanoparticles with the desired shape, size, and distribution is often a challenge. 

In this paper, we introduce the planar density as a broad structural parameter. This parameter involves the effect of both the nanoparticle radius and photonic lattice periodicity. Structures with various spatial distributions of the metallic nanoparticles layer are considered and investigated.

Silver and gold nanoparticles have attracted researchers’ attention in sunlight harvesting by localized surface plasmon [24]. Recently, aluminum nanoparticles have also been of great interest as Al is one of the most abundant elements on earth [25]. Besides, it has favorable compatibility and unique scattering supported with adjustable broad plasmonic resonances [10]. It is, furthermore, a very stable metal, due to the formation of a self-limiting native oxide layer protecting the metal surface from further oxidation and contaminants [24]. In comparison, the detected photocurrent from GaAs photodiodes with gold, silver, and aluminum nanoparticle arrays has been studied [26]. In the blue–green range, Al nanoparticles outperform gold and silver nanoparticles in terms of photodiode maximum efficiency [26]. Although silver outperforms aluminum for the 0.57 to 1.2 µm spectrum, Al is superior for the whole range from 0.3 to 1.2 µm showing a 2.8% gain in the photocurrent [27]. In comparison with thin film silicon solar cells, gallium arsenide solar cells have become a rising star due to higher photoelectric conversion efficiency. Furthermore, the direct bandgap of GaAs makes thin film the active layer enough to use the transmitted light [10].

The design, analysis, and testing of several modern photonic components have been achieved using the finite difference time domain (FDTD) algorithm with second-order numerical accuracy [28,29,30,31]. Several arbitrary geometries with no restriction on the material properties are allowed. Along with experimental endeavors, FDTD simulation improves the productivity of design engineers by saving time and cost plus the better physical interpretation of the mechanisms involved in the considered device [32,33].

This paper is organized as follows: the reported structure and FDTD simulation parameters are described in the Materials and Methods section. The Results and Discussion section represents, in the beginning, the performance characteristics of the bare conventional GaAs thin solar cell followed by the impact of the antireflective coating. Furthermore, the impacts of independently varying both the nanoparticle radius and photonic lattice periodicity are separately considered in simulations and discussion. This section also presents the planar density as a broad structural parameter for the adequate design of the plasmonic nanoparticles layer. Finally, the Conclusion section provides conclusions about the obtained results.

## 2. Materials and Methods

In this work, we comprehensively investigate the optical properties of the thin film plasmonic solar cell by solving Maxwell’s equations of different materials using the FDTD algorithm (OptiFDTD simulation tool from Optiwave Inc., Ottawa, ON, Canada) [34]. 

**Figure 1:**
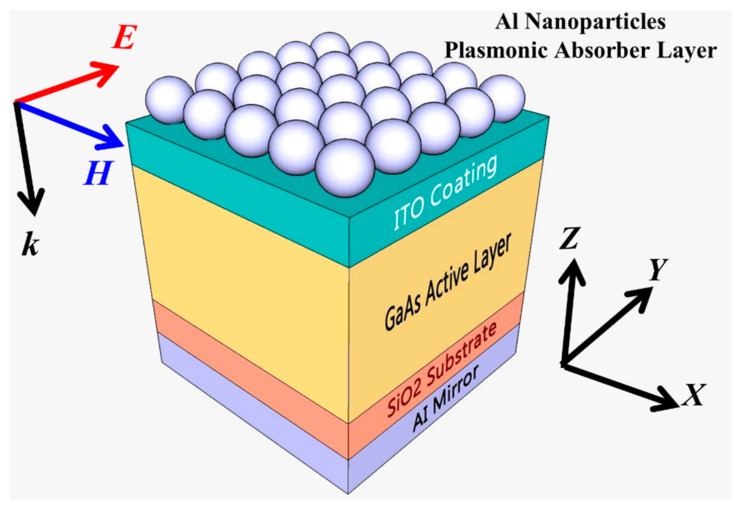
A schematic diagram of thin film GaAs solar cells having Al nanoparticle array placed both on top surface.

The thickness of GaAs active layer is usually optimized for maximizing the conversion efficiency [35]. The trade-off between the intensive bulk recombination and weak thin film absorption should be considered to decide the thickness [36]. Figure 1 shows the schematic of the designed thin film GaAs solar cell having a distinctive random plasmonic Al nanoparticle array on the top. The active material is GaAs layer of thickness 400 nm deposited on a 100 nm silicon dioxide substrate. The active layer was sandwiched between indium tin oxide (ITO) layer on the top and 70 nm aluminum mirror on the bottom. The plasmonic Al nanoparticle array on the top of ITO layer is utilized to boost solar energy harvesting of the thin film GaAs solar cells adjudged by coupling of incident sunlight and plasmonic modes.

The refractive indices for the ITO antireflection coating, GaAs active layer, and SiO_2_ substrate realized in simulations were gathered from the literature [37]. The relative permittivity ε_r_ (ω) of the dispersive Al nanoparticle array and back reflector film was determined using the Lorentz–Drude model [38]:

(1) $$\varepsilon_{r}\left(\omega \right)=\varepsilon_{\infty }+\overset{N}{\underset{m=1}{\sum }}\frac{f_{m}\omega_{om}^{2}}{\omega_{om}^{2}-\omega^{2}+i\omega \Gamma_{m}}$$

where *ε*_∞_ denotes the permittivity at infinite frequency, *f_m_* is a function of the position specifying the oscillator strengths, and *Γ_m_* is the damping coefficient. The incident wave frequency and the resonant frequencies are respectively represented by *ω* and *ω_om_*.

In order to realize a broadband simulation, a linearly polarized Gaussian modulated electromagnetic plane wave was first launched to illuminate the thin film GaAs solar cell at 680 nm center wavelength. The light pulse in time domain has an offset time of 0.8 × 10^−14^ s and half width of 0.1 × 10^−14^ s. Simulation wafer was established in Cartesian coordinates x, y, and z. An anisotropic perfect matching layer was used in the z-direction to avail as absorbing boundary condition, while periodic boundary conditions were used in the x- and y-directions to serve the recurring of the metallic nanoparticles plasmonic layer. The simulation was performed at normal incidence and an x-y observation area 100 nm behind the source was used to calculate the reflection spectrum (R) through the designed solar cell. One can employ the electric field distribution recognized in the simulation waver to calculate the absorption per unit volume in each cell using Equation (2). Then, the absorbed power is normalized by the incident source power [39].

(2) $$P_{abs}=-0.5\;Re\;\left(\mathrm{div}\;S\right)=-0.5\;\omega \;{\left|E\right|}^{2}\;Im\;\left(\varepsilon \right)$$

where **S** denotes the Poynting vector, |**E**| is the magnitude of the electric field intensity on the selected monitor, and *ε* specifies the material dielectric constant. The incident wave frequency is represented by *ω*.

## 3. Results

**Figure 2:**
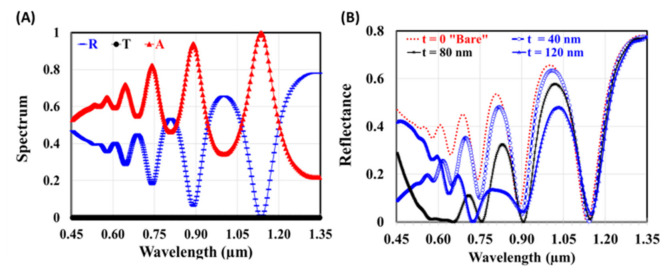
(**A**) Transmittance, reflectance, and absorbance of bare conventional GaAs Scheme (40 nm, 80 nm, and 120 nm); (**B**) Reflectance GaAs solar cell with antireflection coating at different ITO thickness.

Firstly, conventional thin GaAs solar cell, without either an antireflective coating or plasmonic nanoparticle layer, is simulated. Transmittance (T), reflectance (R), and absorbance (A) are displayed in Figure 2A throughout the wavelength window (0.45 to 1.35 µm). The absorbance (A) spectrum was then obtained by A = 1−R, since the transmittance is completely negligible by the thickness of the bottom Al metallic back reflector. Absorption spectra exhibit enhanced crests opposite to highly attenuated troughs in the reflection spectra. Those peaks indicate that when there is perfect matching between the cell structure and free space, less optical energy will be reflected, and consequently optical plasmonic resonance is stimulated. Then, we investigate modifying the reflection spectra of the solar cell by changing the thickness of the ITO antireflective coating layer within a range of 40 to 120 nm with 10 nm step. Figure 2B shows some examples of the reflection spectra of the designed structures at different values of ITO thickness. Thin film anti-reflection coatings greatly reduce the light loss in multi-element lenses by making use of phase changes and the dependence of the reflectivity on the index of refraction.

It is clear that the modulation of the reflectance spectrum, in the visible and near-infrared regions, with the ITO thickness is following the interference condition of Fabry–Perot geometry [40]. On the other hand, changing the thicknesses of the ITO has almost no effect on the reflections at higher wavelengths. We look at the reflections at three different wavelengths 0.48 µm, 0.69 µm, 1 µm, which represent the limits of the visible region and the higher wavelength.

**Figure 3:**
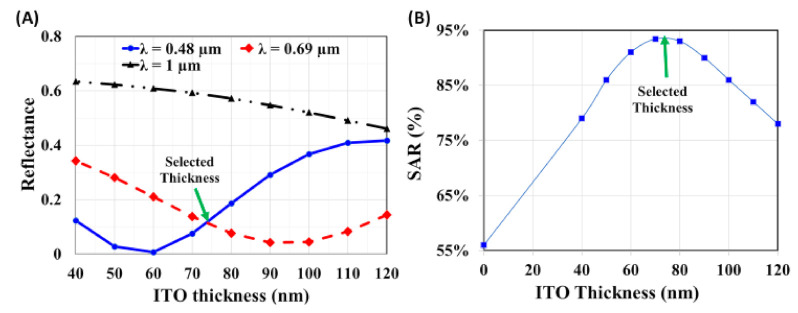
(**A**) Reflectance GaAs solar cell versus the ITO thickness at different incident wavelength (λ = 0.48 µm, 0.69 µm, and 1 µm). (**B**) Impact of changing the ITO thickness on the average spectral absorption rate (SAR).

Figure 3A shows the reflectance spectrum versus different thicknesses of ITO at selected wavelengths. One can select the minima of the intersection of the reflection curves at the limits of the visible region as shown in Figure 3A. The green arrow, shown in Figure 3A, indicates a selected point corresponding to an ITO thickness of 75 nm. At that thickness, the reflection at the edges of the visible region is about 10%.

For further investigation of the effect of the thickness of ITO, the average spectral absorption rate (SAR) is calculated in the visible regime [10]. The SAR can be calculated through a wavelength regime that starts and ends at *λ*_1_ and *λ*_2_ respectively using Equation (3).

(3) $$SAR=\frac{\int_{\lambda_{1}}^{\lambda_{2}}A\left(\lambda \right)d\lambda }{\lambda_{2}-\lambda_{1}}$$

The impact of changing the antireflection coating thicknesses on the optical spectral absorption rate is shown in Figure 3B. The maximum *SAR* occurs at almost 75 nm thickness ITO and confirms our selection.

## 4. Discussion

**Figure 4:**
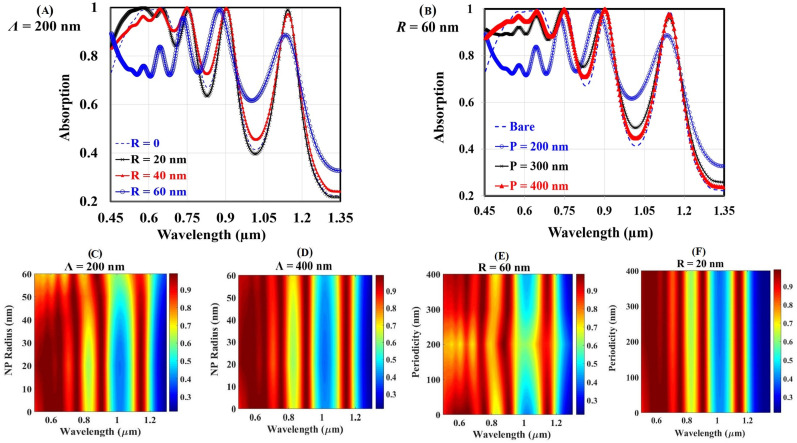
The modification of the absorption spectra due to (**A**,**C**,**D**) changing the Al nanoparticle radius and (**B**,**E**,**F**) changing the structural periodicity.

Secondly, we vary two distinct structural parameters: the nanoparticle radius and photonic lattice periodicity. The impacts of independently varying these parameters are separately considered in our simulations, and some cumulative results are presented in Figure 4.

The modification of the absorption spectra due to changing the Al nanoparticle radius in the range of 20 to 60 nm on the top of the proposed solar cell is illustrated in Figure 4A,C,D. During this investigation, the structural periodicity remains constant at 200 nm in Figure 4A,C and at 400 nm in Figure 4D.

The bare spectrum shows multiple peaks and dips in the near-IR regimes. One may note that adding a nanoparticle layer on the top enhances the absorption and causes some ripples in the near-IR and visible regimes, respectively. For the blue photons, destructive interference of the scattered light may cause absorption quenching. In a higher wavelength regime, the localized surface plasmon resonance of Al nanoparticles enhances the forward scattering and boost the absorption.

Figure 4B,E,F shows the behavior of optical absorption spectra of the reported solar cell structure with changing the structural periodicity. Here, we fix the nanoparticle radius at 60 nm in Figure 4B,E and at 20 nm in Figure 4F.

In the near-infrared (IR) regime, absorption peak enhancement decreases as the period increases. In sharp contrast to this behavior, increasing the structural periodicity decreases the whole absorption spectrum in the visible regime. Inspired by that agonistic behavior in both visible and near-IR regimes due to varying the nanoparticle radius and photonic lattice periodicity independently, the planar density of the Al nanoparticles is introduced as an unchained structural parameter.

**Figure 5:**
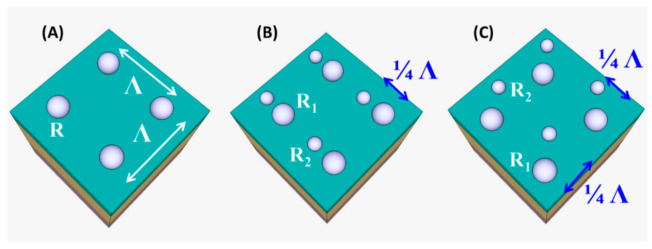
Schematic of the random Al nanoparticle array on top of GaAs solar cell (**A**) Uniform single nanoparticle array (15 different spatial arrangements with various combination of *Λ* = 200 nm, 300 nm, and 400 nm and *R* = 20 nm, 30 nm, 40 nm, 50 nm, and 60 nm. (**B**,**C**) Random double nanoparticle arrays (18 different spatial arrangements with various permutation of *Λ* = 200 nm, 300 nm, and 400 nm, *R*_1_ = 55 nm, 50 nm, 45 nm, and *R*_2_ = 25 nm, 35 nm, 40 nm.

For further investigation, structures with double nanoparticle array of different size and spatial distribution will be introduced as an imperfection from the regular uniform single Al nanoparticle array. Figure 5 depicts the schematic geometry model of the structures used in our investigation.

During this study, the percentage absorption enhancement has been calculated using different structural periodicity (*_Λ_* = 200 nm, 300 nm, and 400 nm). There were 15 different spatial arrangements of uniformly distributed single Al nanoparticle array. The radius of the nanoparticles changes within a range of 20 to 60 nm with a 10 nm step.

Additionally, there were nine different spatial arrangements for each double nanoparticle array. The radius of the first nanoparticle is (*R*_1_ = 55 nm, 50 nm, and 45 nm) while the radius of the second nanoparticle is (*R*_2_ = 25 nm, 35 nm, and 40 nm).

The planar density percentage is calculated as:

(4) $$Planar\;density\;percentage=\frac{\sum \pi R_{i}^{2}}{\Lambda^{2}}\times 100\%$$

The enhancement percentage in the absorption of the structure can be calculated as:

(5) $$Enhancement\;percentage=\;\frac{SAR_{NP}-SAR_{bare}}{SAR_{bare}}\times 100$$

**Figure 6:**
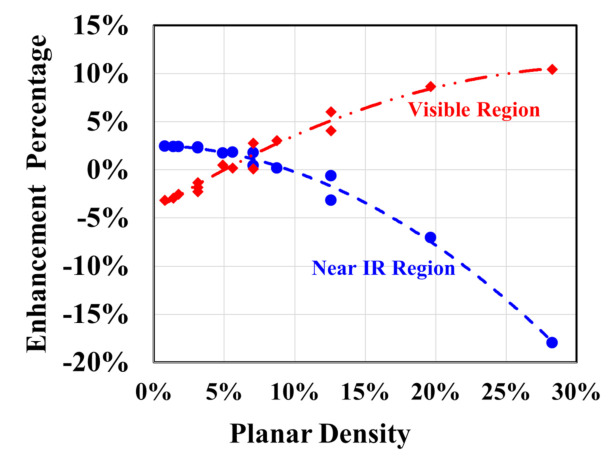
Enhancement percentage in the absorption of the proposed different solar cell structures as a function of the planar density of plasmonic Al nanoparticles layer.

Figure 6 exhibits the enhancement percentage in the absorption of the proposed different structures at different values of the planar density in both the visible and near-IR regimes. As can be seen from this figure, when the planar density is about 7%, the enhancement percentage is identical in both visible and near-IR regimes. However, on increasing the planar density, opposite behavior gradually appears. Increasing the plasmonic planar density enhances the absorption in the visible region. On the other hand, the absorption in the near-IR regime becomes worse, and vice versa. This result is harmonious with the agonistic behavior acquired in Figure 4. The localized surface plasmon resonance of Al nanoparticles enhances the forward scattering and boosts the absorption in the near-IR regime. On the other hand, the destructive interference of the scattered light may cause absorption quenching in the visible regime.

**Figure 7:**
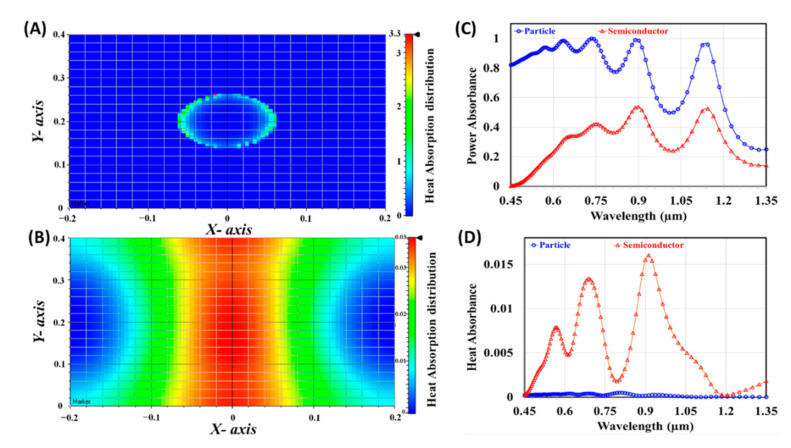
The heat absorption distribution at the center of (**A**) metallic plasmonic layer and (**B**) active semiconductor layer. The (**C**) power and (**D**) heat absorbance spectra at center of both plasmonic and active semiconductor layers.

Finally, the normalized power and microscopic heat absorption are numerically studied and calculated, using two x-y observation slices, at the center of both the metallic plasmonic nanoparticles and semiconductor active layers. Figure 7A,B illustrates the heating absorption distributions in the two x-y observation slices, illustrated in Figure 7A,B.

The scale bars show that the heat absorption captured in the metallic plasmonic layer is higher than that of the semiconductor active layer by about two orders of magnitude. This means the plasmonic nanoparticle is similar to any plasmonic structure that undergoes detrimental absorption, namely the loss of photons inside the metal in the form of heat [41]. The aim is then to minimize this parasitic effect in order to boost the absorption of photons in the active material. The power and heat absorbance spectra at the two x-y monitors are presented in Figure 7C,D, respectively. Although the power absorbed in the front layer of the solar cell is higher, as shown in Figure 7C, most of the heat dissipation occurs in the active layer, as presented in Figure 7D. This means that the active layer absorbs more power, which is favored in optoelectronic devices.

The availability of fabrication techniques as well as the discovery and understanding of plasmonic modes are the key to the success of plasmon research. Synthesis methods must be considered to produce and modulate the desired geometry and optical properties [42]. Additionally, aluminum plasmonics face technical challenges for the manufacture of reproducible structures by simple and low-cost techniques. Recently, adequate deposition parameters that allow great reproducibility of quasi-spherical aluminum nanoparticles using a direct current (DC) sputtering system have been reported [43].

It is also worth mentioning that broadband absorption has been successfully demonstrated using different configurations and structures. About 0.7983 SAR is achieved by incorporation of Al nanoparticles on the top and rear surfaces of the thin-film GaAs solar cell [10]. The Al nanoparticle array decorated on top of the GaAs solar cells has shown a maximum enhanced conversion efficiency of about 1.273 [2].

Regarding the reported structure in this paper, it is very competitive because structural parameters can be changed independently, precisely, and facilely. Furthermore, the planar density is introduced as a broad structural parameter that involves the effect of both the nanoparticle radius and photonic lattice periodicity to tune the sunlight energy harvesting by localized surface plasmons. One can utilize a larger part of the solar spectrum and thus boost the conversion efficiency of solar cells. Inspired by these results and the tandem method, researchers could synthesize the ultimate solar cell.

## 5. Conclusions

In conclusion, we reported the planar density of the plasmonic Al nanoparticle array on the top of a GaAs thin-film solar cell as a broad parameter to tune sunlight energy harvesting. We found that changing the thickness of the antireflective layer and the radius of the plasmonic nanoparticles can enhance the spectral absorption rate from 56 to 95% in the visible region. Furthermore, we present the tuning of the plasmonic resonance through the radius of the nanoparticles to enhance and flatten the absorption spectrum in both the visible and near-IR regimes. Finally, we found that the enhancement percentage in the absorption over the bare structure increases in the visible regime while it decreases in the near-IR regime with a higher planar density of the Al nanoparticle array, and vice versa. Structures with the same plasmonic planar density exhibit identical optical absorption whether in single or double nanoparticle arrays.

## Data Availability

Data available on request from the authors.
